# Identification of pyroptosis-related subtypes, development of a prognostic model, and characterization of tumour microenvironment infiltration in gastric cancer

**DOI:** 10.3389/fgene.2022.963565

**Published:** 2022-07-18

**Authors:** Feng Cao, Jingtao Hu, Hongtao Yuan, Pengwei Cao, Yunsheng Cheng, Yong Wang

**Affiliations:** ^1^ Department of General Surgery, The Second Hospital of Anhui Medical University, Hefei, China; ^2^ Aviation Hygiene Branch, China Eastern Airlines Co,.Ltd, Anhui Branch, Hefei, China; ^3^ Hepatopancreatobiliary Surgery, Department of General Surgery, The First Hospital of Anhui Medical University, Hefei, China

**Keywords:** gastric cancer, programmed death, pyroptosis, tumour microenvironment, immunity

## Abstract

As a new programmed death mode, pyroptosis plays an indispensable role in gastric cancer (GC) and has strong immunotherapy potential, but the specific pathogenic mechanism and antitumor function remain unclear. We comprehensively analysed the overall changes of pyroptosis-related genes (PRGs) at the genomic and epigenetic levels in 886 GC patients. We identified two molecular subtypes by consensus unsupervised clustering analysis. Then, we calculated the risk score and constructed the risk model for predicting prognostic and selected nine PRGs related genes (IL18RAP, CTLA4, SLC2A3, IL1A, KRT7,PEG10, IGFBP2, GPA33, and DES) through LASSO and COX regression analyses in the training cohorts and were verified in the test cohorts. Consequently, a highly accurate nomogram for improving the clinical applicability of the risk score was constructed. Besides, we found that multi-layer PRGs alterations were correlated with patient clinicopathological features, prognosis, immune infiltration and TME characteristics. The low risk group mainly characterized by increased microsatellite hyperinstability, tumour mutational burden and immune infiltration. The group had lower stromal cell content, higher immune cell content and lower tumour purity. Moreover, risk score was positively correlated with T regulatory cells, M1 and M2 macrophages. In addition, the risk score was significantly associated with the cancer stem cell index and chemotherapeutic drug sensitivity. This study revealed the genomic, transcriptional and TME multiomics features of PRGs and deeply explored the potential role of pyroptosis in the TME, clinicopathological features and prognosis in GC. This study provides a new immune strategy and prediction model for clinical treatment and prognosis evaluation.

## Introduction

The 2020 global cancer statistics show that gastric cancer (GC) is the fifth most common cancer, accounting for 5.6% of all cancers worldwide, and has the fourth highest mortality rate (7.7%) (Sung et al., 2021). Due to the insignificant early clinical symptoms and the lack of specific biomarkers, most patients are in the advanced stage of disease at the time of diagnosis. Even though the current routine use of a comprehensive treatment plan based on surgery is supplemented by chemotherapy and immune-targeted therapy, the 5-years survival rate of patients with advanced GC is still only approximately 30% (Montagnani et al., 2018). In recent years, an increasing number of studies have suggested that this phenomenon may be related to the inhibition of tumour cell programmed death and tumour microenvironment (TME)-mediated immune heterogeneity (Yan et al., 2018; Tang et al., 2020). Therefore, in-depth analysis of the genes and immune microenvironment characteristics of tumour cell programmed death is the key to identifying different patient subgroups, understanding the potential pathogenesis of GC, exploring precise therapeutic targets and predicting the effect of immunotherapy.

Pyroptosis, also known as cytoinflammatory necrosis, is a gasdermin-mediated programmed cell death characterized by continuous cell swelling until the cell membrane ruptures, thereby releasing cellular contents, including interleukin (IL)-18, IL-1β, high mobility group box 1 (HMGB1) and proinflammatory factors such as adenosine triphosphate (ATP), which in turn activate a strong inflammatory response. Its mechanism mainly relies on the inflammasome recruiting proteins of the caspase family (caspase-1/4/5/11), cleaving and activating the gasdermin protein and translocating it to the cell membrane to form a hole, thereby leading to cell pyroptosis (Shi et al., 2015). A large number of studies have shown that pyroptosis plays an important role in the occurrence and development of various tumours, including GC. For example, Tan G et al. (Tan et al., 2020). Found that gasdermin E (GSDME) can promote the occurrence of colorectal cancer by mediating the pyroptosis of intestinal epithelial cells and releasing HMGB1. Wang WJ et al. (Wang et al., 2018a). Also found that low expression or inhibited expression of gasdermin D (GSDMD) can promote tumour cell proliferation in GC. Zhang X et al. (Zhang et al., 2021). Showed that the *H. pylori* cytotoxin-related gene A (CagA) protein can activate the NOD-like receptor protein 3 (NLRP3) inflammasome pyroptosis pathway and promote GC cell migration and invasion. In addition, pyroptosis also plays an important role in the molecular targeted therapy of tumours. For example, Chunfeng Li et al. (Li et al., 2021) found that low-dose diosbulbin-B (DB) can activate tumour-inherent programmed cell death one ligand 1 (PD-L1)/NLRP3 signalling pathway-mediated inflammatory cell death, thereby increasing cisplatin sensitivity in GC. However, the current research on pyroptosis in GC is limited to this, and there is a lack of research on the relationships between related genes and the prognosis and clinical characteristics of GC patients.

The TME is a complex environment in which tumour cells survive and develop. It is mainly composed of an immune microenvironment dominated by immune cells and a nonimmune microenvironment dominated by fibroblasts, including tumour cells, inflammatory cells, endothelial cells, and various cytokines. A large number of studies have shown that the TME plays a key role in multiple tumour pathological processes, such as tumorigenesis, local drug resistance, immune escape and distant metastasis. Studies by Vitale et al. (Vitale et al., 2019) have shown that tumour-associated macrophages (TAMs) in the TME can account for more than 50% of some solid tumours and are involved in tumour progression and resistance to therapy. Similarly, tumour-associated fibroblasts (CAFs) also play an important role in the TME. For example, Monteran et al. (Monteran and Erez, 2019) showed that CAFs can promote tumour cell growth, angiogenesis and immune escape through multiple pathways. The same is true in GC: Li W et al. (Li et al., 2019) found that GC-derived mesenchymal stromal cells (GC-MSCs) could promote GC cell metastasis by inducing the polarization of M2 macrophages. Miao et al. (Miao et al., 2020) reported that the proinflammatory subtype of TAMs can activate the IL6R/JAK/IL24 signalling pathway to induce apoptosis in GC cells. In addition, tumour-infiltrating cells within the TME can also predict the survival prognosis of tumour patients (Cai et al., 2020). However, due to the complexity, variability and high heterogeneity of the TME, many mechanisms are still unclear.

Therefore, comprehensive analysis of the genomic, transcriptional and TME multiomics features of pyroptosis-related genes (PRGs) and in-depth exploration of the potential role of pyroptosis in the TME, clinicopathological features and prognosis in GC are crucial to providing new immune strategies and predictive models for clinical treatment and prognosis evaluation.

## Materials and methods

### Data sources and preprocessing

The workflow chart of this study is shown in Supplementary Figure S1. The Gene Expression Omnibus (GEO) https://www.ncbi.nlm.nih.gov/geo/) and The Cancer Genome Atlas (TCGA) (https://portal.gdc.cancer.gov/) databases were used to obtain the GSE84437, GSE38749, and TCGA GC cohorts for downloading GC gene expression (fragments per kilobase million, FPKM), gene mutation, and associated prognostic and clinical data, and the raw files obtained were also normalized and annotated. Among them, All patients included in this study had no history of radiotherapy and chemotherapy, and all tissue samples were primary tumors. The “limma” package of the R language was used to convert the FPKM value of the TCGA data into the TPM value. Then, the three datasets were integrated using the “limma” and “sva” packages, and after excluding patients with a lack of OS data, a total of 886 GC patients were obtained for subsequent analysis.

### Analysis of genetic and transcriptional alterations of PRGs in GC

We obtained a total of 56 PRGs from MSigDB Team (REACTOME_PYROPTOSIS) (http://www.broad.mit.edu/gsea/msigdb/) and previous publications (Liberzon, 2014; Xia et al., 2019; Zhou and Fang, 2019; Fang et al., 2020; Ye et al., 2021). Combined with the mutation data of TCGA, mutation analysis was performed using the “maftools” package of the R language, and a waterfall chart of PRG mutations was created. In addition, we downloaded the GC copy number data from UCSC Xena (https://xena.ucsc.edu/) and used “Rcircos” of the R language to analyse the frequency of PRG copy number variation (CNV) and the chromosomal location of CNV genes.

### Differential expression and consensus clustering analysis of PRGs

The expression of PRGs in the GC dataset was extracted by using the R language “limma” package, and then the “reshape2” and “ggpubr” packages were used to analyse the data and create the expression difference map of PRGs in tumour samples and normal samples. The clinical data from the GSE84437, GSE38749, and TCGA GC datasets were integrated, and the survival curves and interaction networks of differentially expressed PRGs were plotted using the R language. According to the expression level of PRGs, patients were divided into different molecular subtypes by consensus unsupervised cluster analysis by the k-means method using the R language “ConsensusClusterPlus” package.

### Relationship between pyroptosis-related subtypes and the clinical features and prognosis of GC

To test the clinical value of the two pyroptosis-related subtypes identified by consensus unsupervised clustering, we used R language analysis to compare the differences in clinicopathological features and prognosis between the two subtypes. Among them, clinicopathological features included age, sex, TNM stage and tumour clinical stage, and the difference in prognosis was assessed by Kaplan-Meier survival curves.

### Gene set variation analysis and immune cell infiltration

We used the R language “GSVA” package and downloaded the “c2.cp.kegg.v7.4.symbols” pathway set file from the MSigDB database to complete the GSVA enrichment analysis and display it with a heatmap. In addition, a single-sample gene set enrichment analysis (ssGSEA) algorithm was used to quantitatively analyse each cell to compare differences in immune cell infiltration among different subtypes (Rooney et al., 2015).

### Analysis of DEGs and functional annotation

We continued to use R language analysis to obtain differentially expressed genes (DEGs) between the two pyroptosis-related subtypes with a fold-change of 1.5 and an adjusted *p* value of <0.05. Gene Ontology (GO) and Kyoto Encyclopedia of Genes and Genomes (KEGG) functional enrichment analyses were performed.

### Prognosis-related gene subtype identification and differential analysis

To screen genes related to tumour prognosis, the R language “survival” package was used to perform univariate Cox regression analysis on DEGs, and all prognosis-related DEGs were included in the next model construction. Consensus unsupervised cluster analysis was performed on these genes to classify patients into different genotypes. Likewise, survival analysis and clinicopathological analysis were performed between the different subtypes.

### Prognosis model construction and validation

First, GC patients were randomized 1:1 into training and test cohorts using the “caret” package. Second, using the R package “glmnet” to incorporate prognostic-related DEGs into the least absolute shrinkage and selection operator (LASSO) and Cox regression analysis, we analysed the change trajectory of each independent variable and used 10-fold cross-validation. Then, the risk score (RS) was calculated with genes whose regression coefficients were not zero. The RS was calculated as $RS=\sum_{j=1}^{n}Xj*Coefj$ , where n represents the number of included prognosis-related DEGs and Xj and Coefj represent the expression level and risk coefficient of each prognosis-related DEG, respectively. The training and test cohorts were divided into low-risk and high-risk groups based on the median risk score. Survival differences between the two risk groups in the training and test cohorts were assessed using the Kaplan-Meier method and log-rank test. Finally, the time-dependent receiver operating characteristic (ROC) curve was plotted using the “SurvivalROC” package, and the area under the curve (AUC) was calculated to assess the ability of RS to predict prognosis.

### Establishment of a nomogram scoring system

First, we performed univariate and multivariate Cox regression analyses on RS and clinical characteristics, demonstrating that RS was an independent factor predicting prognosis. Then, the “rms” package was used to build a predictive nomogram. This scoring system assigns a value to each variable and calculates the sum of all variables for each sample to assess the probability of OS at 1, 3, and 5 years. Among them, the calibration curve was used to evaluate the accuracy of the nomogram in predicting prognosis.

### Analysis of immune cell infiltration, TME, MSI, and CSCs

To explore the degree of immune cell infiltration in each sample, we calculated the relative content of various immune cells using the “preprocessCore” and “e1071″ packages and used the CiberSort algorithm to correlate various immune cells with RS and prognosis-related DEG analysis. Then, we analysed the tumour microenvironment (TME) and further explored tumour purity by calculating the stromal score, immune score and estimation core in each tumour sample using the “estimate” package (Yoshihara et al., 2013). In addition, we also performed correlation analysis between microsatellite instability (MSI) and cancer stem cells (CSCs) in the high-and low-risk groups.

### Mutation and drug susceptibility analysis

We first used the “maftools” package to analyse the somatic mutations in samples from the GC high-and low-risk groups. Then, tumour mutational burden (TMB) scores and correlation coefficients with RS were calculated for each sample in both groups. Finally, to further explore the differences in the sensitivity of chemotherapeutic drugs between the high-and low-risk groups, we installed the pRophetic software package to calculate the semiinhibitory concentration (IC50) values of commonly used chemotherapeutic drugs and set the filter condition to *p* < 0.001.

### Statistical analyses

All statistical analyses and integration were performed using R version 4.1.2 and Strawberry Perl version 5.32.1.1. *p* < 0.05 was considered statistically significant.

## Results

### Genetic changes and expression variations of PRGs in GC

**FIGURE 1:**
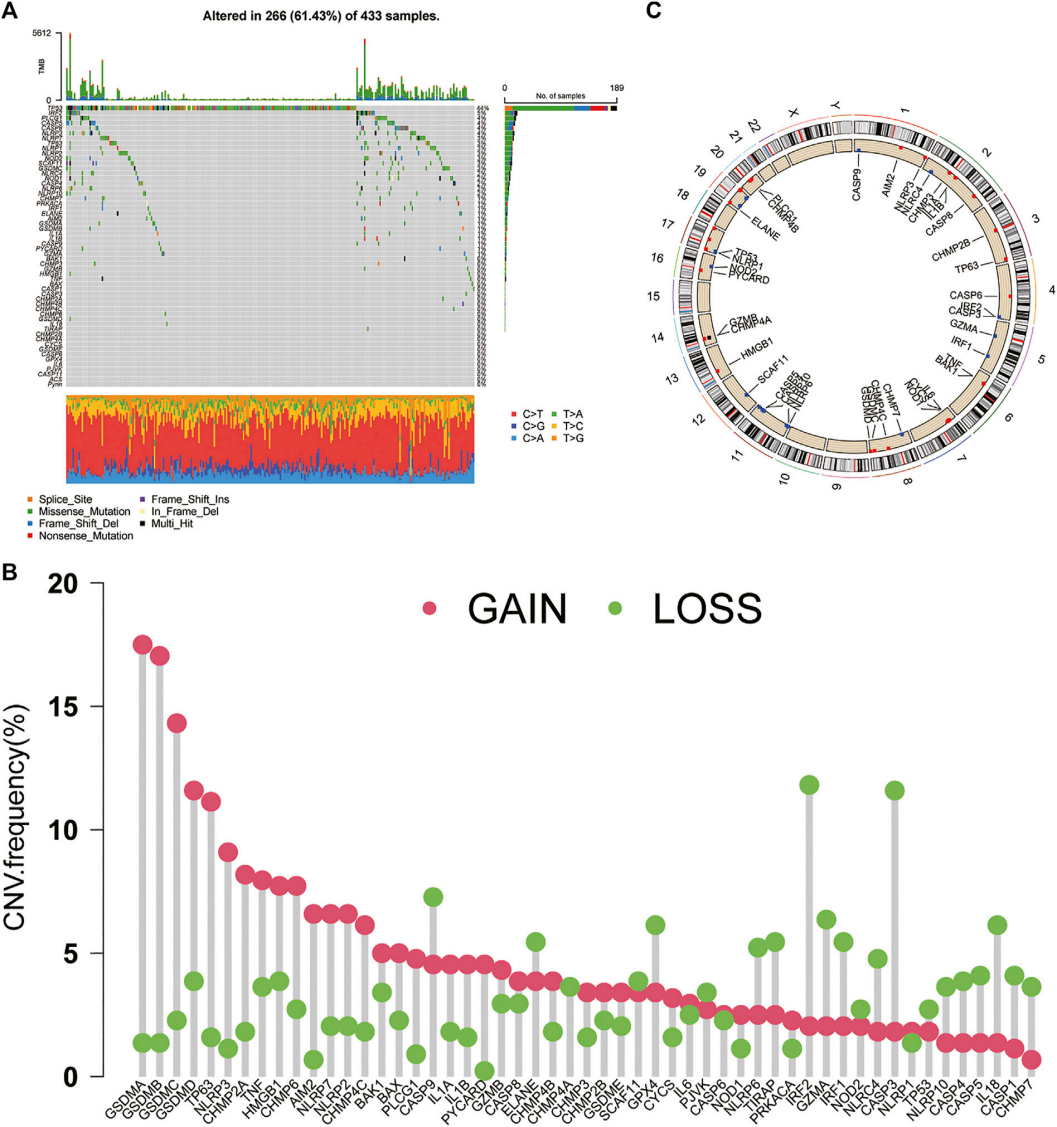
Genetic and transcriptional alterations of PRGs in GC **(A)** Mutation frequencies and types of PRGs in 433 samples in TCGA **(B)** CNV of PRGs in 433 samples in TCGA **(C)** Chromosomal localization of PRGs with CNV. PRGs: pyroptosis-related genes. CNV: copy number variation.

A total of 56 PRGs were included in this study. Somatic mutation analysis of these PRGs revealed relatively high mutation frequencies in the GC cohort. Among the 433 samples, 266 samples had PRG mutations, and the mutation frequency was as high as 61.43%. Among them, a total of 30 PRGs were mutated, and the highest mutation frequency of TP53 was 44%, followed by IRF2, PLCG1, CASP5/8, NLRP3/7, etc. In addition, waterfall plots showed the highest frequency of missense mutations and G-to-T transitions in single nucleotide polymorphisms (SNPs) in these mutated PRGs (Figure 1A). We investigated the copy number alterations of PRGs and found that CNVs are prevalent in PRGs, including unmutated PRGs. Among them, the four proteins A/B/C/D of the GSDM protein family had the highest CNV frequencies, and the frequency of amplification was significantly higher than that of deletion. In contrast, CNVs of IRF2, CASP3, CASP9, GZMA, and IL-18 were extensively absent (Figure 1B). Additionally, in Figure 1C, the types of CNVs that occur in PRGs can also be found. In addition, the figure also shows their respective positions on the chromosome (Figure 1C).

### Identification of prognosis-related PRGs and pyroptosis-related subtypes

**FIGURE 2:**
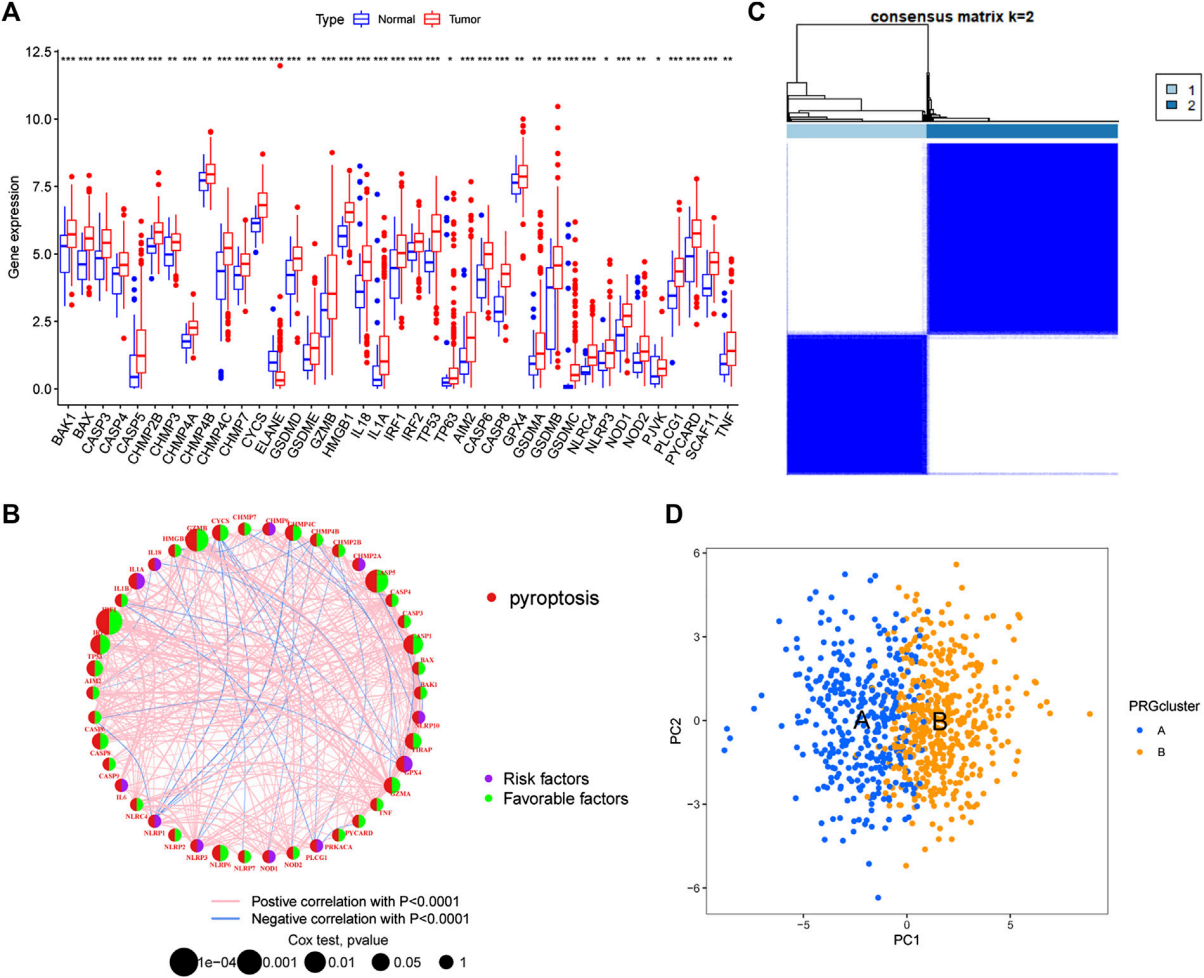
Identification of pyroptosis-related subtypes **(A)** Differential expression of PRGs between tumour tissues and normal tissues **(B)** PRG interaction network **(C)** Consensus unsupervised clustering defines two clusters (*k* = 2) and their correlation area **(D)** PCA divided GC samples into two subtypes. PCA: principal Component Analysis.

We further compared the expression levels of PRGs between tumour tissues and normal tissues in GC and found that 39 PRGs were differentially expressed. Except for ELANE, which was expressed at low levels in tumour tissues, the remaining genes were highly expressed in tumours (Figure 2A). In addition, combined CNV analysis revealed that PRGs with higher CNV frequencies had more significant differences in expression, especially the GSDM protein family. However, there are also PRGs (CHMP2A, CHMP6, NLRP2, GPX4, and GZMA, etc.) that develop CNV but do not differ in their expression levels. Therefore, we speculate that PRGs serve a potential role in the molecular mechanism of the occurrence and development of GC, and that their expression levels may be regulated by CNVs. Next, we performed univariate Cox regression and Kaplan-Meier survival analysis based on PRG expression and clinical data and found that 32 PRGs were associated with GC prognosis (Supplementary Figure S2). The PRG interaction network results further showed that IRF1, GZMB, and CASP5 were most associated with prognosis (Figure 2B). Interestingly, these genes also had the highest frequency of CNV deletions. This finding indicates that CNV may be an influencing factor of GC prognosis. Finally, we classified GC patients by consensus unsupervised cluster analysis of PRGs (Supplementary Figure S3). The results showed that *k* = 2 was the best choice, with the highest sample correlation within the A and B subtypes (Figure 2C). In addition, PCA also confirmed this result; that is, GC samples can be divided into two subtypes, A and B, according to the expression levels of PRGs (Figure 2D).

### Differences in clinical characteristics in distinct subtypes and functional annotations of DEGs

**FIGURE 3:**
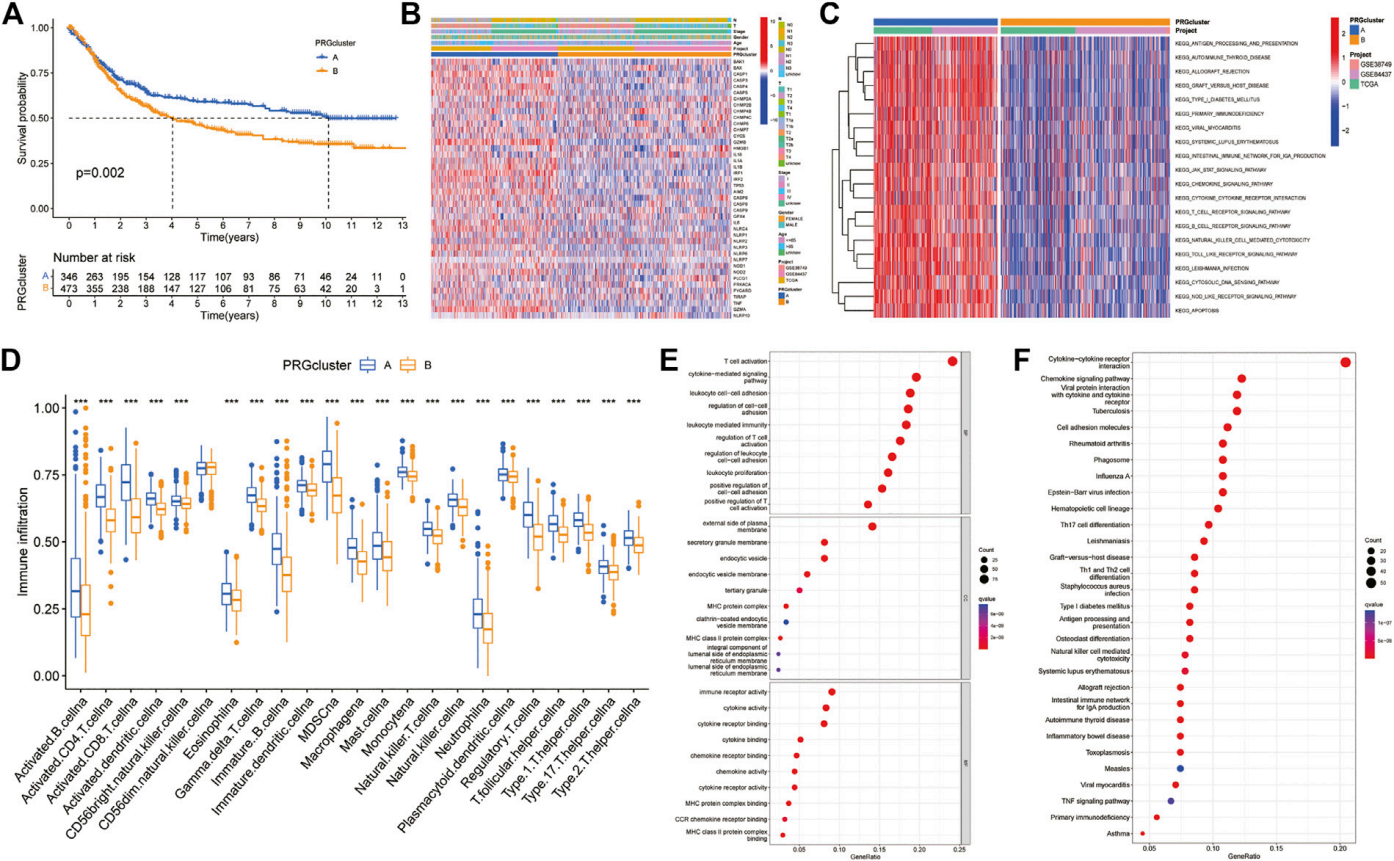
Difference analysis between two PRG-related subtypes **(A)** Kaplan-Meier survival curves between the two subtypes **(B)** Differences in clinical characteristics in distinct subtypes **(C)** GSVA enrichment analysis **(D)** Differences in immune cell infiltration between the two subtypes **(E,F)** GO and KEGG enrichment analyses of pyroptosis-related DEGs. DEGs: differentially expressed genes. GO: Gene Ontology. KEGG: Kyoto Encyclopedia of Genes and Genomes.

The results of survival difference analysis between different subtypes showed that the Kaplan-Meier survival curve of subtype A was significantly better than that of subtype B, indicating that patients with subtype A had a longer OS than patients with subtype B (Figure 3A). In addition, the analysis of clinicopathological characteristics showed that compared with the B subtype, PRGs were expressed at a higher level in the A subtype, and the TNM stage and clinical stage of the A subtype were lower than those of the B subtype (Figure 3B). Moreover, GSVA enrichment analysis revealed that subtype A was significantly enriched in antigen processing and presentation, T and B-cell receptor signalling pathways, natural killer cell-mediated cytotoxicity, cytokine and chemokine signalling pathways, the JAK/STAT signalling pathway, the NOD-like and Toll-like receptor signalling pathways, and apoptosis, as well as immune-related pathways (Figure 3C). Next, we further compared the immune infiltration between the two subtypes, and the results showed that for all immune cells (activated B cells, activated CD4 T cells, immature dendritic cells and type 1 T helper cells), infiltration scores were significantly different between the subtypes, and the immune infiltration scores were higher in subtype A than in subtype B (Figure 3D). Finally, 422 DEGs between different subtypes were obtained using the R language “limma” package, and corresponding functional annotation revealed that DEGs were mainly enriched in biological processes such as the regulation of T-cell and leukocyte activation and adhesion, as well as in immune-related molecular functions such as immune receptors and cytokine and chemokine activities (Figure 3E). Kyoto Encyclopedia of Genes and Genomes (KEGG) analysis revealed that DEGs were mainly enriched in immune-related pathways such as cytokine signalling, differentiation of T-cell subtypes, and natural killer cell-mediated cytotoxicity (Figure 3F). Therefore, these results suggest that pyroptosis plays an important role in immune regulation.

### Identification of DEG subtypes and construction of a prognostic model

**FIGURE 4:**
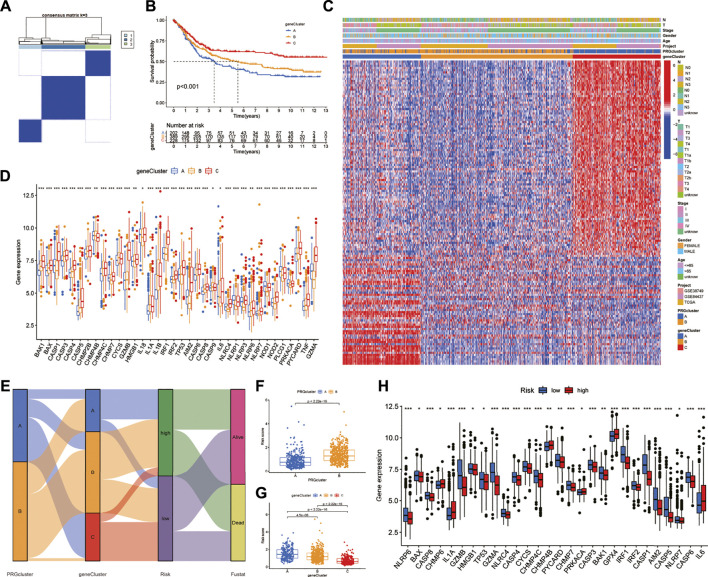
Identification of DEG subtypes and construction of the prognostic model **(A)** Consensus unsupervised clustering defines two clusters (*k* = 3) and their correlation area **(B)** Kaplan-Meier survival curves between the three DEG-related subtypes **(C)** Differences in clinical characteristics between the three DEG-related subtypes **(D)** Expression differences of PRGs between the three DEG-related subtypes **(E)** Alluvial diagram of subtype distributions in groups with different PRG scores and survival outcomes **(F,G)** Differences in RS with respect to different phenotypes **(H)** Differential expression of PRGs in the high-and low-risk groups. RS: risk score.

Univariate Cox regression analysis showed that 141 of 422 DEGs were associated with prognosis. The consensus unsupervised clustering analysis of the prognosis-related DEGs found that *k* = 3 was the best choice, and these prognosis-related DEGs could be divided into three subtypes (Figure 4A). Kaplan-Meier survival curves showed that subtype A had the worst prognosis and subtype C had the best prognosis (Figure 4B). In the analysis of clinicopathological features, it was also found that subtype A was associated with advanced TNM stage and clinical stage, which may be related to the downregulation of DEGs upregulated in subtype C and downregulated in subtype A (Figure 4C). In addition, the differential expression analysis of PRGs showed that there were 36 differentially expressed PRGs in the three genotypes. Among them, CYCS and IL-1BZ were highly expressed in the B subtype, NOD1, PLCG1, and PRKACA were highly expressed in the A subtype, and the remaining PRGs were highly expressed in subtype C (Figure 4D).

We randomly divided GC patients into training (*n* = 409) and test (*n* = 409) groups. Then, LASSO regression analysis was performed on the 141 prognostic DEGs, the risk coefficient of each DEG was calculated and obtained, and 17 prognosis-related DEGs were retained according to the minimum partial likelihood deviance (Supplementary Figures S4A,B). Finally, nine prognosis-related DEGs were obtained according to multivariate Cox regression analysis, and RS was calculated according to their risk coefficients (Table 1). Patients in the training and test groups were divided into low-risk and high-risk groups according to the median RS value. The Sankey diagram shows the process of constructing the prognostic model and the distribution of GC patients in different subtypes (Figure 4E). In addition, the results of RS difference analysis showed that in the two pyroptosis-related subtypes, the RS of subtype A with better prognosis was significantly lower than that of subtype B. Likewise, among the three DEG subtypes, RS had the highest value in the worst-prognosis subtype A and the lowest value in the best-prognosis subtype C (Figures 4F,G). These results confirmed that RS may be one of the factors affecting OS in GC patients. In addition, there were differences in the expression of PRGs in the high-and low-risk groups. CHMP6, IL-1A, CHMP4B, PRKACA, GPX4, and IL-6 were highly expressed in the high-risk group, while 22 genes such as NLRP6, GZMB, CASP1, GZMA, and AIM2 were highly expressed in the low-risk group (Figure 4H).

**TABLE 1:** Risk coefficients of nine prognosis related DEGs.

Id	Risk coefficients
IL18RAP	−0.225900268,311,302
CTLA4	−0.186986822,788,874
SLC2A3	0.128274018,078,394
IL1A	0.191851618,279,267
KRT7	0.1018325,630,033
PEG10	0.0883859924,715,283
IGFBP2	0.10334699,006,446
GPA33	−0.109104140,756,844
DES	0.048614134,175,904

### Validation of the prognostic model and development of a nomogram

**FIGURE 5:**
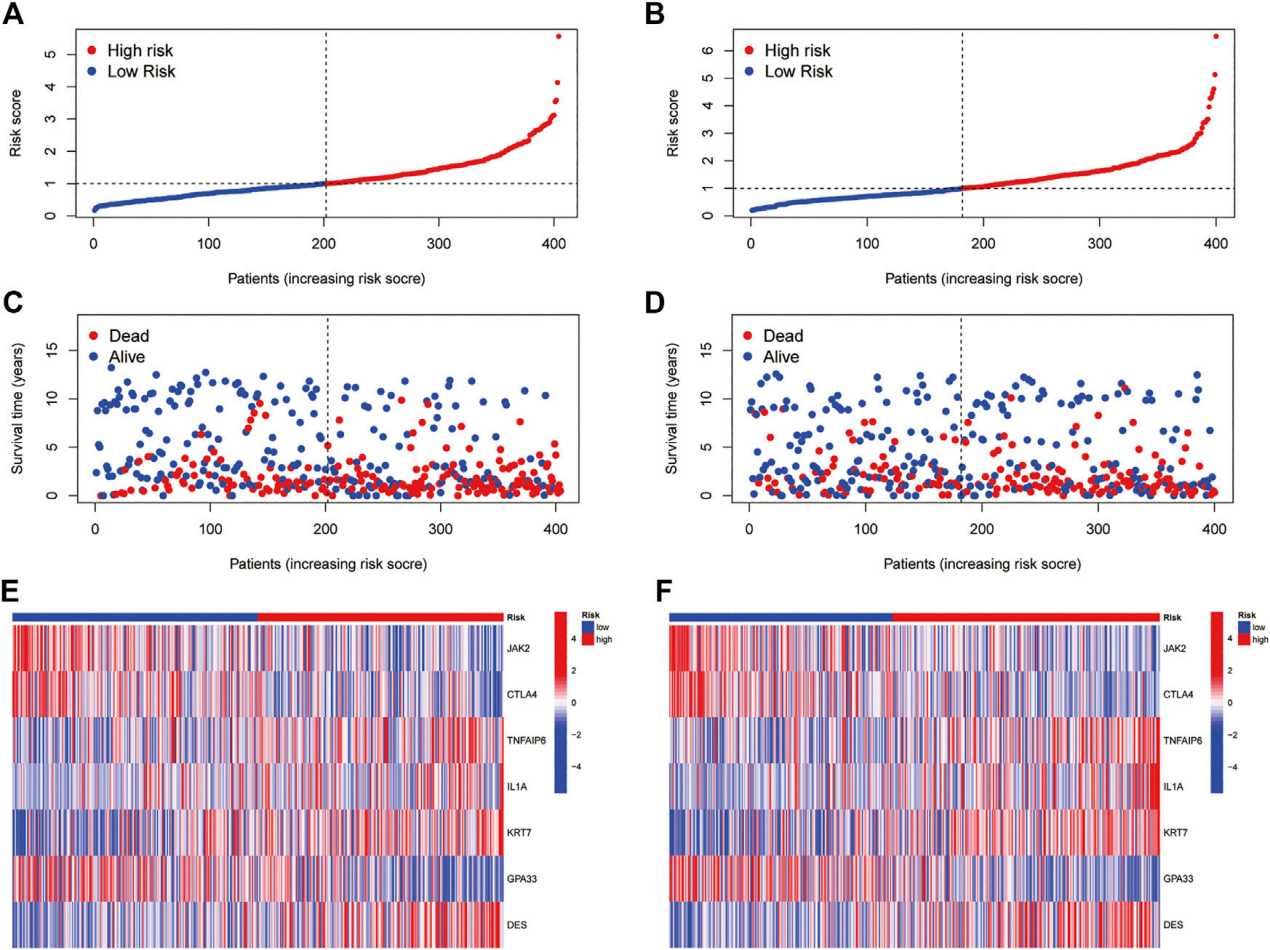
Prognostic value of the pyroptosis-associated DEG signature in the training and test cohorts **(A,B)** RS distribution **(C,D)** Survival status of GC patients **(E,F)** Heatmap of the 7 DEGs.

**FIGURE 6:**
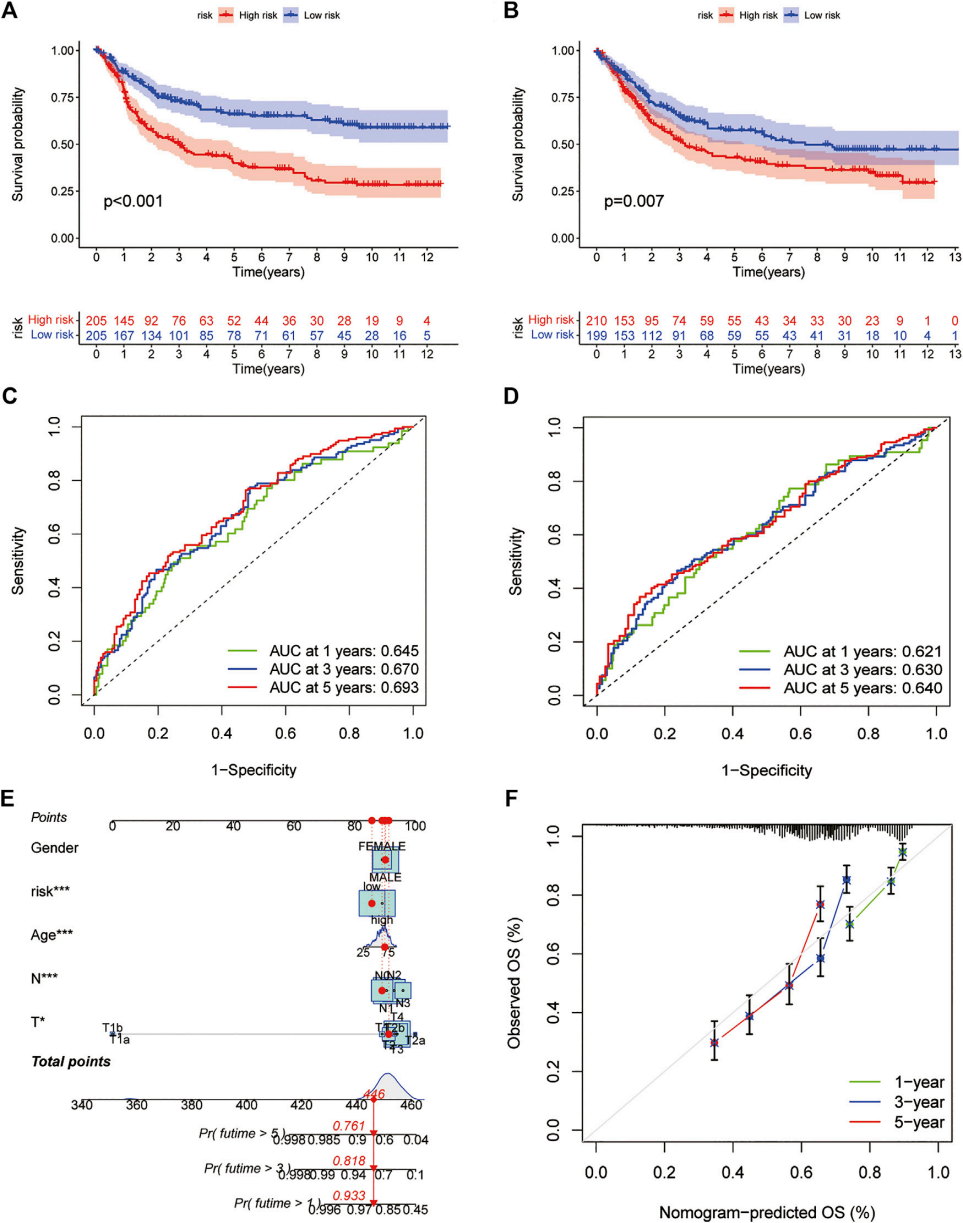
Prediction model **(A,B)** Kaplan-Meier survival curves between the high- and low-risk groups in the training and test cohorts **(C,D)** ROC curves predicting 1-, three- and 5-years OS in the training and test cohorts of GC patients **(E)** Nomogram for predicting the 1-, three- and 5-years OS of GC patients **(F)** Calibration curves of the nomogram. ROC: receiver operating characteristic.

All GC patients were divided into low-risk and high-risk groups according to the median RS value, the RS of each patient was calculated according to the prognostic model, and the patients in the training and test groups were ranked in sequence (Figures 5A,B). Analysing the OS of patients, it was found that the mortality rate of patients in the high-risk group in the training group was significantly higher than that in the low-risk group, and this phenomenon was also consistently observed in the test group (Figures 5C,D). The heatmaps of prognosis-related DEGs in both groups indicated that TNFAIP6, IL-1A, KRT7, and DES were highly expressed and JAK2, CTLA4, and GPA33 were expressed at low levels in the high-risk group (Figures 5E,F). In addition, in both the training group and the test group, the Kaplan-Meier survival curve showed that the survival rate of GC patients in the high RS group was significantly lower than that in the low RS group (Figures 6A,B). To evaluate the accuracy of our prognostic prediction model, using ROC curve analysis, the area under the curve (AUC) values of the training group at 1, 3, and 5 years of OS were 0.645, 0.670, and 0.693, respectively. Similarly, the test group also had good accuracy; that is, the AUCs at 1, 3, and 5 years were 0.621, 0.630, and 0.640, respectively (Figures 6C,D). Therefore, this study created a nomogram using clinical features and RS to predict OS in GC patients (Figure 6E). At the same time, the calibration diagram also confirmed the reliability of the model in predicting OS at 1, 3, and 5 years (Figure 6F).

### Correlation of RS with immune cells and differences in TME and MSI between the high-and low-risk groups

**FIGURE 7:**
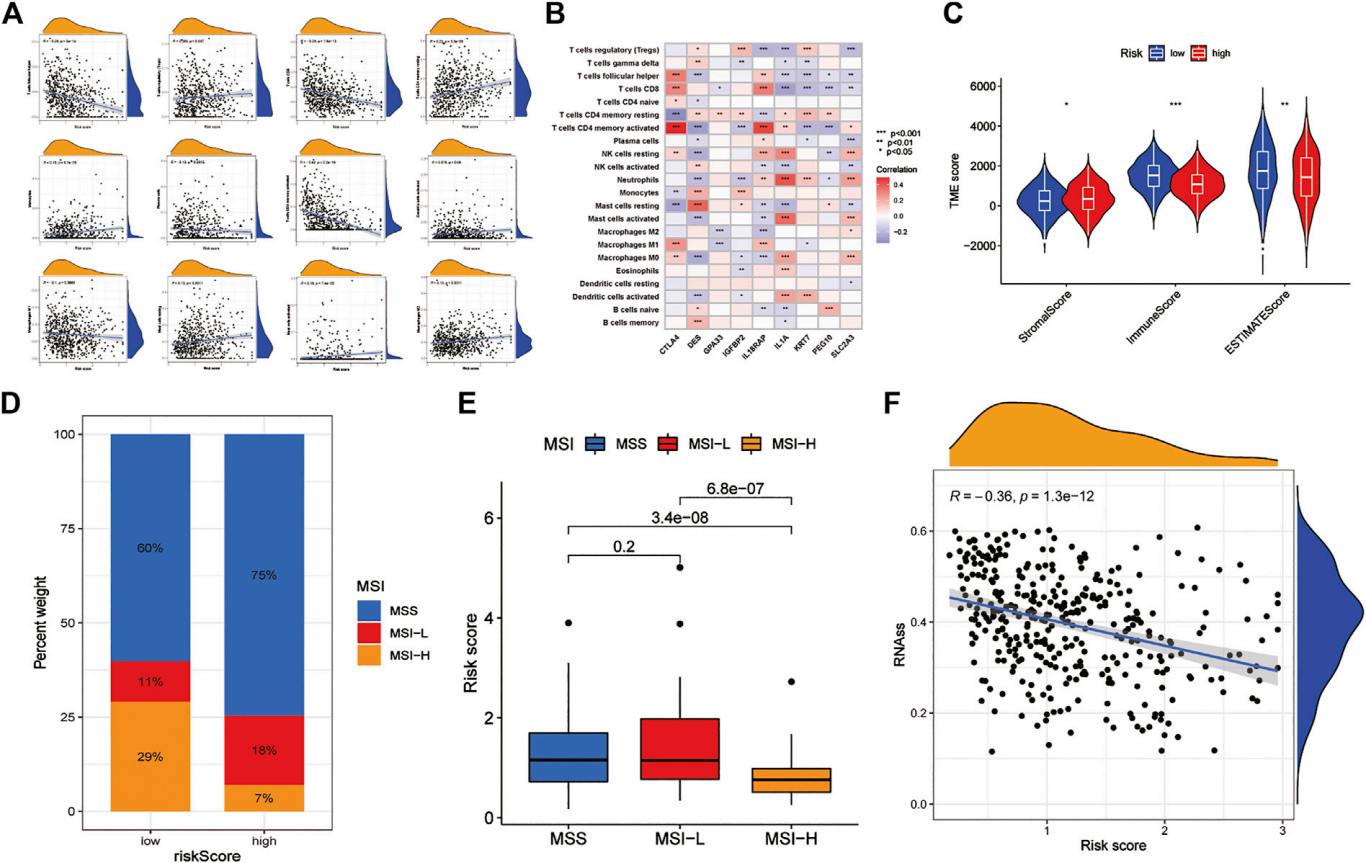
Analysis of TME characteristics between the high- and low-risk groups **(A)** Correlation between immune cells and RS **(B)** Correlations between the abundance of immune cells and 9 DEGs **(C)** Differences in the StromalScore and ImmuneScore between the high- and low-risk groups **(D,E)** Relationships between RS and MSI **(F)** Correlation of RS with CSC. TME: tumour microenvironment. MSI: microsatellite instability. CSC: cancer stem cells.

Correlation analysis between RS and immune cell abundance showed that RS was positively correlated with T regulatory cells, activated dendritic cells, M1 macrophages, M2 macrophages, activated mast cells, resting mast cells, monocytes, neutrophils, plasma cells, and resting memory CD4^+^ T cells and inversely correlated with activated memory CD4^+^ T cells, CD8^+^ T cells, and follicular helper T cells (Figure 7A). In addition, the nine prognostic DEGs in the prognostic model were also significantly associated with multiple immune cells (Figure 7B). In addition, TME difference analysis revealed differences in the StromalScore, ImmuneScore and ESTIMATEScore between the low-risk and high-risk groups. Among them, the low-risk group had lower stromal cell content, higher immune cell content and lower tumour purity (Figure 7C). Furthermore, the results of MSI difference analysis showed that RS was significantly correlated with MSI status. The proportions of microsatellite stability (MSS) and low microsatellite instability (MSI-L) in the low-risk group were significantly lower than those in the high-risk group, and high microsatellite instability (MSI-H) was significantly higher than that in the high-risk group (Figure 7D). These results are consistent with the RS differences among MSS, MSI-L and MSI-H (Figure 7E). This suggests that patients in the low-risk group are more sensitive to immunotherapy. Finally, the correlation results between RS and CSCs showed that RS and CSCs were significantly negatively correlated, indicating that GC cells with lower RS exhibited more poorly differentiated stem cell characteristics (Figure 7F).

### Mutation and drug susceptibility analysis

**FIGURE 8:**
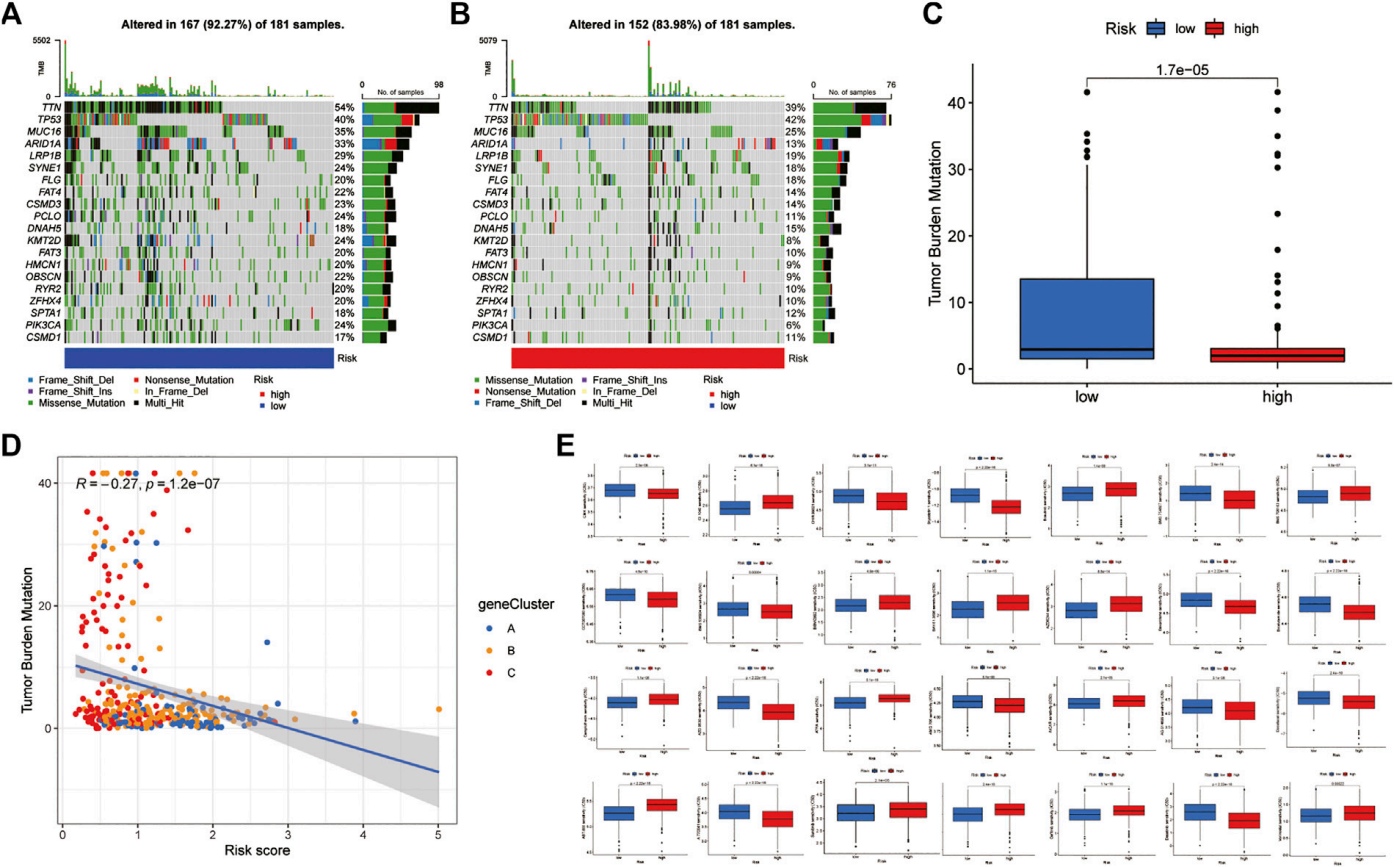
Mutation and drug susceptibility analysis **(A,B)** Mutation and drug susceptibility analysis **(C,D)** Relationships between RS and TBM **(E)** Relationships between RS and chemotherapeutic sensitivity. TMB: tumour mutational burden.

The somatic mutation analysis between the low-risk and high-risk groups of GC patients found that the top 20 mutated genes in the two groups were TTN, TP53, MUC16, ARID1A, LRP1B, SYNE1, FLG, FAT4, CSMD3, PCLO, DNAH5, KMT2D, FAT3, HMCN1, OBSCN, RYR2, ZFHX4, SPTA1, PIK3CA, and CSMD1. However, the mutation frequency of samples in the low-risk group was significantly higher than that in the high-risk group. Among them, the mutation frequency of the rest of the genes was lower than that of the low-risk group, except that the mutation frequency of TP53 in the high-risk group was slightly higher than that in the low-risk group (Figures 8A,B). In addition, the correlation analysis between RS and TMB found that the TMB score in the low-risk group was significantly higher than that in the high-risk group and was significantly negatively correlated with RS (Figures 8C,D). This is consistent with the results of the MSI analysis indicating that patients in the low-risk group may have better immunotherapy outcomes. Finally, drug sensitivity analysis found that RS was associated with drug sensitivity. There were significant differences in the IC50 values of various chemotherapeutics, including afatinib, veliparib, motesanib, saracatinib, selumetinib, bexarotene, bicalutamide, bosutinib, camptothecin, dasatinib, docetaxel, gefitinib, etc., between the low-risk and high-risk groups (Figure 8E).

## Discussion

In recent years, studies have shown that pyroptosis plays an indispensable role in the occurrence and development of tumours and has strong immunotherapy potential, but the specific pathogenic mechanism and antitumor function are still unclear, especially in GC research, which is even more scarce (Hsu et al., 2021). Therefore, this study reveals the multiomics characteristics of mutations, CNV, expression profiles, TME and immune infiltration of PRGs in GC by comprehensively analysing the overall alterations in genomic, transcriptional and TME levels in GC, deeply explores the potential role of cellular scoring in the immune microenvironment, clinicopathological features and prognosis of GC, and provides new immune strategies and drugs for clinical treatment targets.

The results of this study showed that PRGs had a higher frequency of missense mutations and CNVs in GC, and among them, TP53, IRF2, PLCG1, and CASP5/8 had the highest mutation frequencies. TP53, a DNA-binding tumour suppressor protein, is mutated in more than 50% of malignant tumours, including human hereditary cancers such as Li-Fraumeni syndrome (Baugh et al., 2018). There are two main methods of single base substitution and allele loss which mediate the inactivation of viral or intracellular tumour suppressor proteins, thereby promoting the occurrence and development of cancer (Tommasino et al., 2003). Numerous studies have shown that single-base substitutions are one of the major forms of mutation in the entire coding sequence (Olivier et al., 2010). Consistent with the results of this study, SNPs were second only to missense mutations in mutated PRGs, and the frequency of base G-to-T conversion was the highest. Their different types and locations may be the key to revealing the molecular mechanisms of cancer occurrence and development and may also serve as potential diagnostic and prognostic markers and targets for drug intervention. Therefore, IRF2, PLCG1 and CASP5/8 mutations may also be key targets for GC progression. Studies have found that interferon regulatory factor 2 (IRF2) can improve colorectal cancer responsiveness to PD-1 therapy by directly inhibiting chemokine 3 (CXCL3) (Liao et al., 2019). Likewise, phospholipase C gamma 1 (PLCG1) and caspase 5/8 (CASP5/8) also exert antitumor effects in cancer (Li et al., 2015; Kim et al., 2020). In addition, as a 1 KB-3MD DNA fragment variation, CNVs are widely distributed in the human genome. The total number of nucleotides covered by CNVs far exceeds that of SNPs, and CNVs larger than 250 kb are closely related to tumour susceptibility and phenotype (Zarrei et al., 2015). Gasdermin (GSDM) proteins, as key effector molecules of pyroptosis, are frequently amplified in cancer genomes and exert antitumor effects through cell death-mediated protein inactivation. However, some studies have suggested that GSDMs can not only exert an antitumor effect but also serve a tumour-promoting function, but this depends on the cellular environment, especially the interaction between cancer cells and the tumour microenvironment (Sarrio et al., 2021). Hou J et al. (Hou et al., 2020) found that PD-L1 can promote tumour cell death by mediating GSDMC expression. In addition, we also found that the genes (GSDM, IRF1, GZMB, and CASP5, etc.) with a higher frequency of CNV have higher expression differences between normal and tumour tissues and greater correlation with prognosis, which further highlights the key role of CNV in GC. Therefore, we believe that gene mutations, CNVs and SNPs may be the main factors affecting the function of PRGs in tumours, and that related gene functions are also affected by the TME.

This study further constructed the risk model for predicting prognostic and screened nine PRGs related key genes for exploring new immunotherapy strategies and drug targets. The results showed that the model had high accuracy in predicting OS in GC patients. Compared with the models constructed by previous studies, This study is the first to construct a pyroptosis-related signature in GC. Surprisingly, The accuracy of the pyroptosis related signature in predicting the 1, 3, and 5-years OS of GC patients was significantly higher than that of the ferroptosis related signature constructed by Jiang (Jiang et al., 2021). Beisdes, we also deeply explored the signature’s characteristics in TME, and found that the risk score of this model was significantly correlated with immune infiltration, TME, MSI, and CSCs. This indicates that key genes in the model may play an important role in the clinical features and prognosis of GC (Xiang et al., 2021; Zheng et al., 2021).

Among them, cytotoxic T lymphocyte associated protein 4 (CTLA4) is a member of the immunoglobulin family, expressed on the surface of activated T cells, and is homologous to CD28, both of which can bind to CD80 and CD86 on the surface of antigen presenting cells, thereby reducing T cell proliferation and cytokine production in GC (Dovedi et al., 2021). Therefore, studies have found that the use of CTLA-4 antibody can induce the expansion of tumor-infiltrating CD 8 + T cell Th1-like CD 4 + T cell subsets (Wei et al., 2017). Pereira et al. (Pereira et al., 2021) also confirmed that positivity for both CTLA-4 and PD-L1 was an independent factor associated to better survival in GC patients. In addition, although desmin (DES) is expressed in smooth muscle tissue, it has been found to be more sensitive in the detection of vascular invasion in gastric, colorectal and pancreatic cancers (Ekinci et al., 2018). Glycoprotein A33 (GPA33) is a cell-surface diferentiation glycoprotein and a member of the immunoglobulin superfamily. Although its specific function is currently unclear, studies have found that GPA33 is expressed in more than 95% of colorectal cancers and about 70% of GCs, but not in normal epithelial cells (Garinchesa et al., 1996; Sakamoto et al., 2000). This indicates that GPA33 may be a diagnostic marker and potential therapeutic target for GC and colorectal cancer. This view was confirmed by Lopes et al. (Lopes et al., 2020). High expression of caudal related homeobox transcription factor 2 (CDX2) and its target GPA33 has favorable effects on GC prognosis. Insulin like growth factor binding protein 2 (IGFBP2) is the second most abundant IGFBP in tissues and exerts biological functions by regulating IGF receptors. The expression in GC tissue is significantly higher than that in normal mucosa, which can promote the proliferation of GC cells by activating the IGF1R-RhoA-ROCK signaling pathway (Zhang et al., 2007; Liu et al., 2022). Interleukin 18 Receptor Accessory Protein (IL18RAP) and Interleukin 1A (IL-1A) are inflammatory factors of the same family. Studies have found that their polymorphism increases the susceptibility of *Helicobacter* pylori-induced inflammation and carcinogenesis of the gastric mucosa (Durães et al., 2014; Wang et al., 2016). In-depth exploration of the expression differences of different genotypes of these genes in gastric cancer will provide strategies for the drug treatment of GC. Keratin 7 (KRT7), a member of the keratin family, is abnormally expressed in a variety of tumor tissues and has been widely accepted as a prognostic marker for GC. KRT7 and his antisense in orientation to coding mRNA (KAT7-AS) were significantly upregulated in GC tissues, promoting GC cell proliferation and migration (Huang et al., 2016). In addition, Liu et al. (Liu et al., 2019) also confirmed this view and showed that KAT7 reduced the sensitivity of 5-fluorouracil treatment. Paternally expressed gene 10 (PEG10) also has the function of promoting the proliferation of GC cells. But the difference is that PEG10 is also a key gene for chemotactic lymphocytes (Ishii et al., 2017). The family gene of solute carrier family two member 3 (SLC2A3) is a central regulator of cellular glucose metabolism, but its role in tumor metabolism and immunity has been neglected. Studies have found that SLC2A3 not only promote the aerobic glycolysis of GC cells, but also promote the M2 subtype transition of macrophages in the TME (Yao et al., 2020). This well explains why SLC2A3 is positively correlated with M2 macrophages in the results of this study.

In addition, patients of subtype A with high PRG expression had a better prognosis and relatively higher immune infiltration scores and signalling pathways mainly enriched in immune-related pathways such as antigen processing and presentation, T and B-cell receptor signalling pathways and apoptosis. Furthermore, the low-risk group had a lower content of stromal cells, a higher content of immune cells and a lower tumour purity. This indicated that a large number of immune cells infiltrated low-purity tumour tissue, including immune-promoting cells and immune-suppressing cells. This finding suggests that PRGs may play an important role in GC through the tumour immune microenvironment (TIME). Studies have shown that increased CD8^+^ density on the surface of T cells in tumours and immune stroma is associated with an increased percentage of PD-L1 expression (Thompson et al., 2017). While quiescent T cells lacked PD-1 expression on the surface, high levels of PD-1 expression were detected in tumor-infiltrating T cells. The indicated that immune induced tumor PD-1 expression may be a way for tumor cells responsed to immunotherapy resistance mechanisms. Interestingly, CTLA4 is predominantly intracellular in resting naive T cells and expressed on the surface of activated T cells. However, GC cells can inhibit the metabolism of CD8^+^ T cells through the CD155/TIGIT signalling pathway, thereby inhibiting the antitumor immune response (He et al., 2017). Traditional immunization believes that CD8^+^ T cells induce tumor cells death through two pathways, perforin-granzyme and Fas-FasL. But the latest research has discovered a new mechanism by inducing ferroptosis and pyroptosis. Studies have shown that pyroptosis-related genes or RNAs are involved in the occurrence and development of multiple tumors, and have great potential for the diagnosis, treatment and prognosis prediction of GC (Xu et al., 2020; Wu et al., 2022). However, activation of the cell death program leads to the release of inflammatory mediators IL1A and IL18RAP, which continue to promote cancer development. Wang et al. (Wang et al., 2020). Found that pyroptosis and immune checkpoint inhibitors act synergistically, and the inflammatory response can trigger a strong antitumor immune function by establishing a bioorthogonal chemical system. This conclusion was also confirmed by Zhang et al. (Zhang et al., 2020), who showed that in the TIME activated by pyroptosis, CD8^+^ T cells and NK cells can mutually induce and activate tumour cell pyroptosis through granzyme B and form a positive feedback loop. Besides, Ji L et al. (Ji et al., 2020). Found that inhibit Treg cell infiltration by blocking CCL28 can effectively inhibit GC progression. This indicates that Treg plays the opposite role in the tumor microenvironment, and has the ability to promote the immune escape of tumor cells and indirectly accelerate the proliferation and infiltration of tumor cells. Treg cells are also the main cells for CTLA4 expression and are negatively regulated by CTLA4.

Moreover, the degree of infiltration of B cells in GC, especially memory B cells and plasma cells, also seriously affects tumour progression and prognosis (Ni et al., 2021). Some studies have found that B-cell translocation gene 3 (BTG3) is significantly downregulated in GC, which may be related to tumour proliferation, migration, and invasion, and has potential as a biomarker for predicting the prognosis of GC patients (Ren et al., 2015). In addition, tumor-associated macrophages (TAMs) can exhibit different activation states according to the stimulation of different TMEs. M1 macrophages mainly secrete proinflammatory factors such as IL-1α, IL-1β, and TNF12, which positively activate antitumor responses (Pan et al., 2020). M2 macrophages secrete some anti-inflammatory factors, such as IL-4, IL-6, and IL-10, which negatively regulate the immune response in tumours (Sousa et al., 2015). Interestingly, increased numbers of cytokine IL-4, growth factor colony-stimulating factor-1 (CSF-1) and CD4^+^ T cells in the TME stimulate the macrophage M1 to M2 transition (Lin et al., 2002; Mantovani and Sica, 2010; Coussens et al., 2013). M2 macrophages had immunosuppressive and contribute to matrix remodeling, thereby promoting tumor growth. There are high levels of matrix metalloproteinase-9 in GCs, which degrade collagen in type IV basement membranes, thereby promoting metastasis (Li et al., 2019). Previous studies have shown that M2 macrophages are involved in Epithelial mesenchymal transition (EMT), and that infiltration of M2 macrophages in the TME enhanced GC transfer (Liang et al., 2019; Lin et al., 2019). Furthermore, high tumor stromal density of M2 macrophages have been associated with worse cancer-specific survival in patients, and patients with high M1:M2 density ratio in tumor stroma have a higher survival rate.

The proportion of MSI-H in the low-risk group was significantly higher than that in the high-risk group, and RS was negatively correlated with both CSC and TMB. According to some studies, MSI occurs in approximately 15% of early colorectal cancer tissues. Among them, MSI-H is closely related to clinicopathological characteristics and prognosis, such as tumour location and clinical stage (Gelsomino et al., 2016). Similarly, Vilar E (Vilar and Gruber, 2010) and Yang et al. (Yang et al., 2019) agreed with this view and stated that the MSI phenotype is associated with specific oncogenes and tumour suppressor genes, such as BRAF, MRE11A, and KRAS. The MSI phenotype is expected to become an important marker for evaluating the degree of tumour malignancy and predicting efficacy and prognosis. In addition, in this study, CSC and TMB were also important factors for good prognosis of GC patients in the low-risk group. CSCs have the ability to self-renew and differentiate, which is a decisive factor in tumour heterogeneity (Prasetyanti and Medema, 2017). Moreover, studies have shown that CSCs are also closely related to tumour occurrence and treatment resistance (Eun et al., 2017). Finally, this study also screened potential GC therapeutic drugs through drug sensitivity analysis. Among them, paclitaxel and cisplatin can effectively inhibit tumour proliferation and metastasis by inducing pyroptosis (Wang et al., 2018b; Zhang et al., 2019). Studies have shown that exogenous activation of pyroptosis can produce strong antitumor activity, especially in digestive system tumours, which are abnormally sensitive (Wei et al., 2014; Wang et al., 2019). Therefore, inducing tumour cell pyroptosis may provide a new direction for targeted drug research and tumour therapy.

## Conclusion

In conclusion, this study revealed mutations, CNVs, and TME multiomics characteristics of PRGs by comprehensively analysing the overall changes in the genomic and transcriptional levels of PRGs in GC and constructing a prognosis prediction model for GC patients. We found IL18RAP, CTLA4, SLC2A3, IL1A, KRT7, PEG10, IGFBP2, GPA33, and DES are key genes of gastric cancer and deeply explored the potential role in clinicopathological features, immune infiltration, TME and prognosis in GC, providing new immunotherapy strategies and drug targets for the clinical treatment of GC patients.

## Data Availability

The original contributions presented in the study are included in the article/Supplementary Material, further inquiries can be directed to the corresponding authors.
