# Supplementary material for: Identification of pyroptosis-related subtypes, development of a prognostic model, and characterization of tumour microenvironment infiltration in gastric cancer
Source: Front Genet. 2022 Jul 18;13:963565. doi: 10.3389/fgene.2022.963565 (PMC9340157; doi:10.3389/fgene.2022.963565)
Supplement: Supplementary file 1 [file DataSheet1.docx]

**Identification of pyroptosis-related subtypes, development of a prognostic model, and characterization of tumour microenvironment infiltration in gastric cancer**

**Data declaration**

All original data have been uploaded to Jianguoyun (https://www.jianguoyun.com/p/De0BwosQ9r_FChjao70EIAA).

**Supplementary Figure**


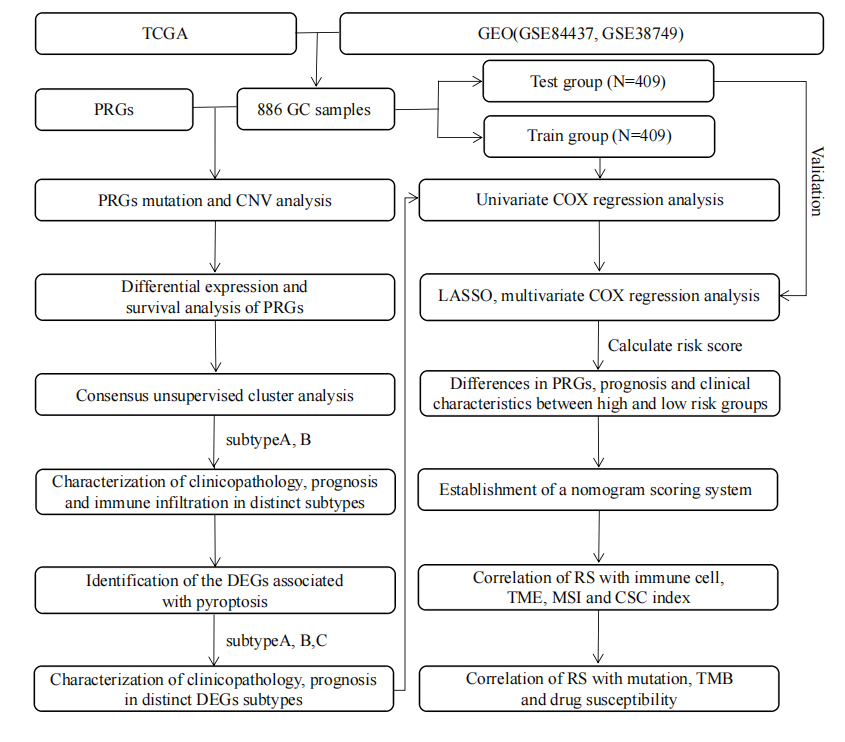


**Supplementary Figure 1** The entire analytical process of the study.


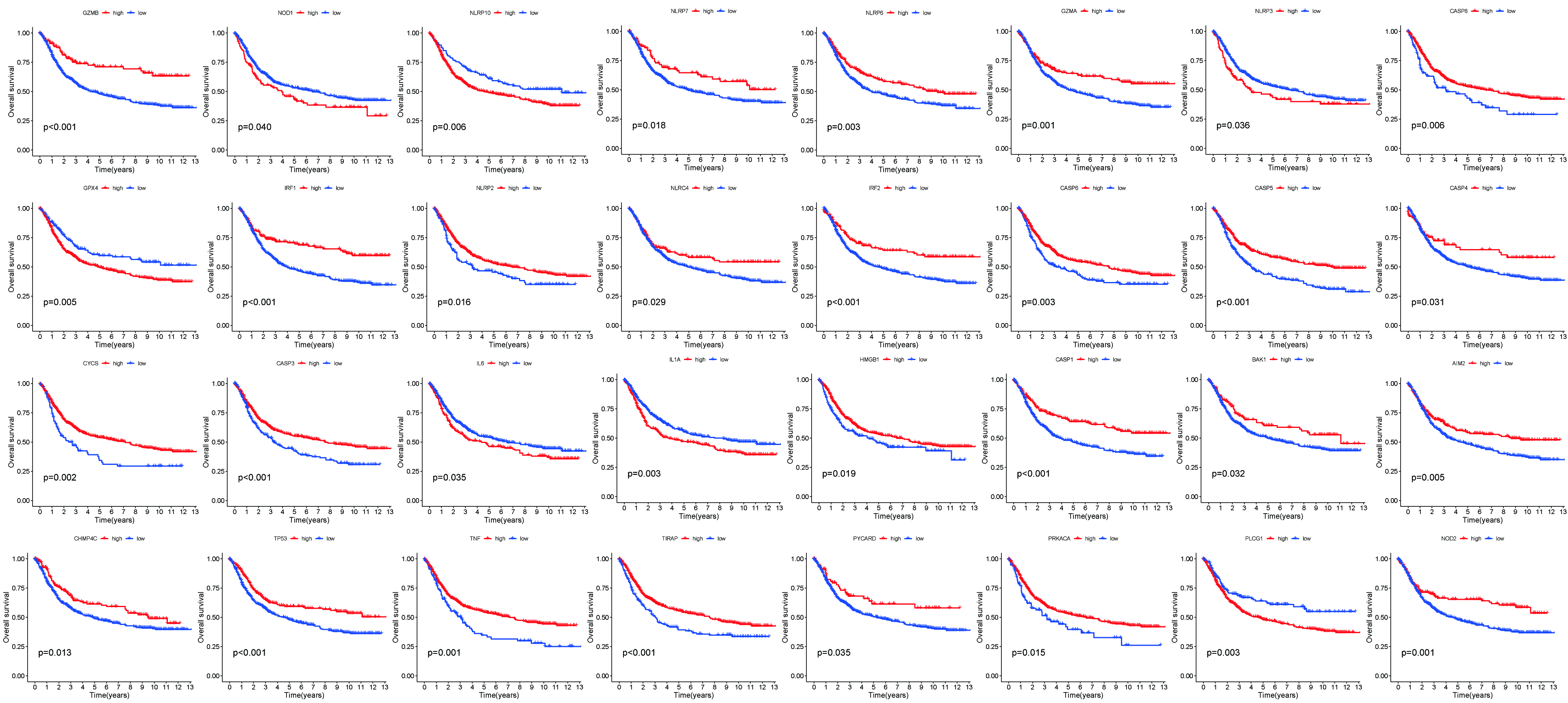


**Supplementary Figure 2** Kaplan–Meier survival analysis of 32 PRGs associated with GC prognosis.


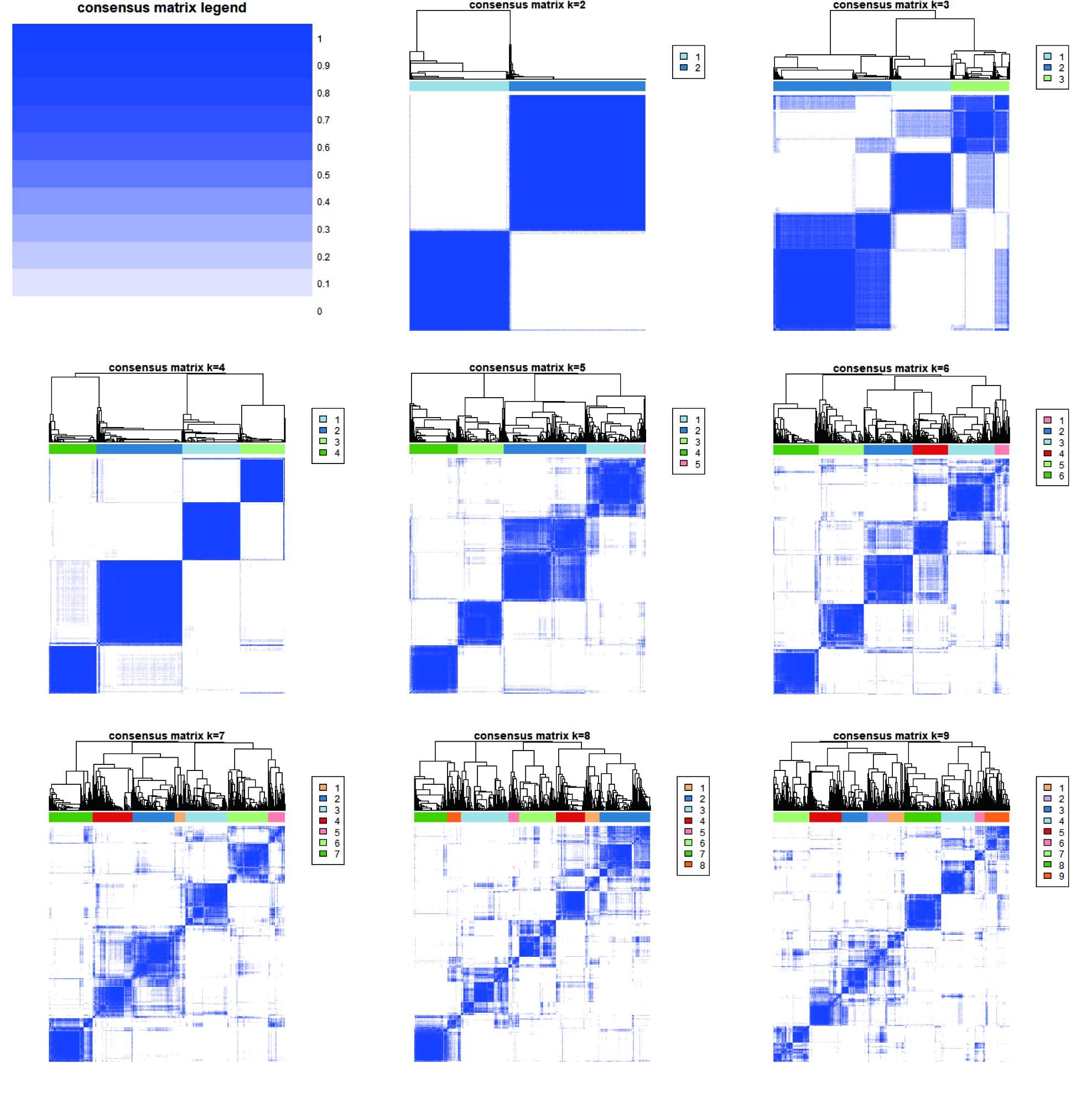


**Supplementary Figure 3** Unsupervised clustering of pyroptosis-related genes and consensus matrix heatmaps for k = 3-9.


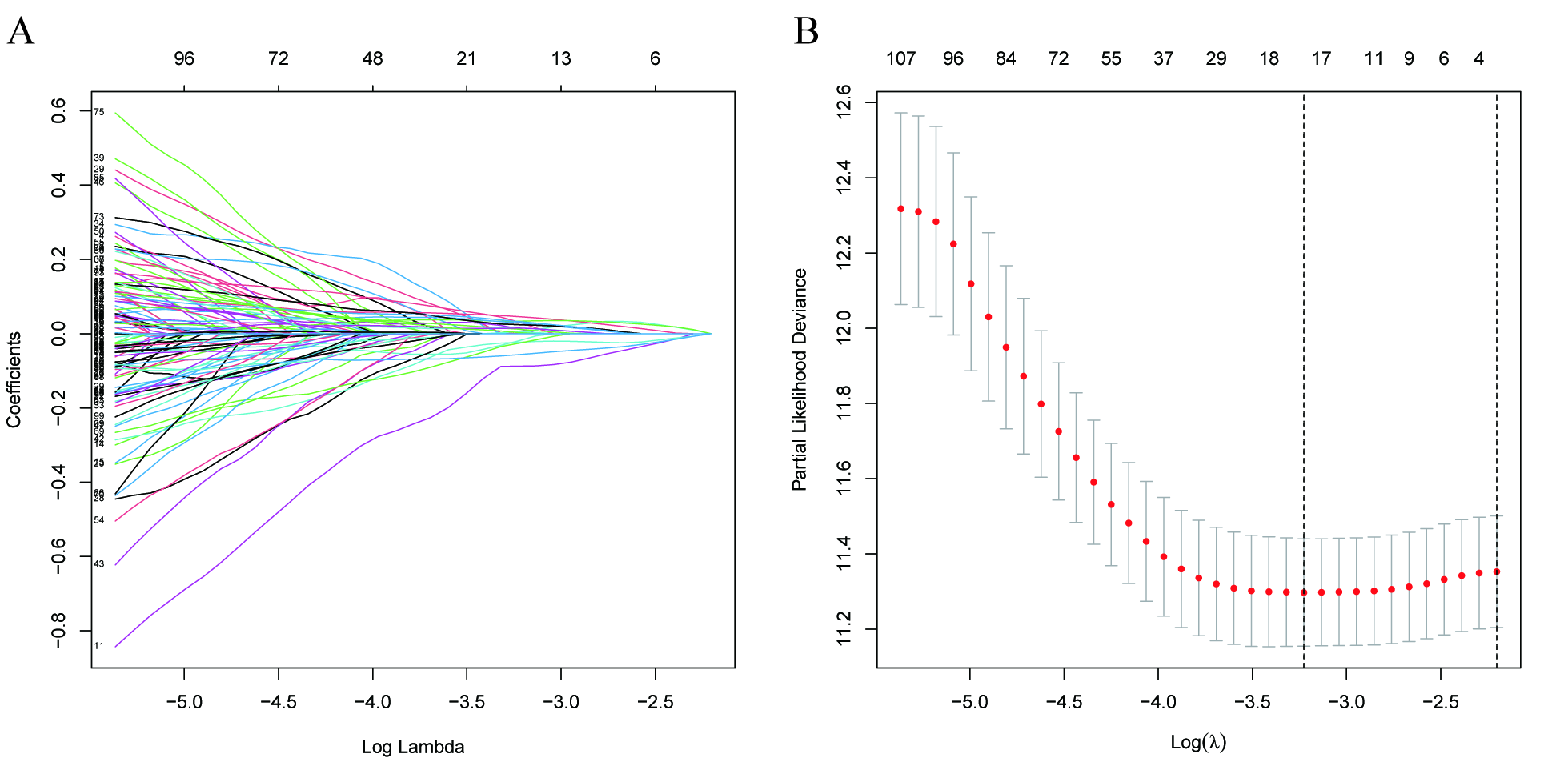


**Supplementary Figure 4** Identifying representative candidate prognostic DEGs. (A-B) LASSO regression analysis and partial likelihood deviance of the prognostic DEGs.
